# Study protocol for measuring stigmatization in persistent tic disorders: development and validation of the Tourette discrimination-stigmatization scale

**DOI:** 10.3389/fpsyg.2024.1381063

**Published:** 2024-04-30

**Authors:** Jaclyn M. Martindale, Victor M. Ringheanu, Kelly A. Pring, Sandra Norona, Kimberly Wiseman, Roy E. Strowd, Leah Chapman, Joseph Rigdon, Stephen R. Rapp, Eric A. Storch, Edward Ip, Jonathan W. Mink

**Affiliations:** ^1^Department of Neurology, Wake Forest University School of Medicine, Winston-Salem, NC, United States; ^2^Wake Forest University School of Medicine, Winston-Salem, NC, United States; ^3^Clinical and Translational Science Institute, Wake Forest University School of Medicine, Winston-Salem, NC, United States; ^4^Department of Social Sciences and Health Policy, Wake Forest University School of Medicine, Winston-Salem, NC, United States; ^5^Department of Biostatistics and Data Science, Wake Forest University School of Medicine, Winston-Salem, NC, United States; ^6^Department of Psychiatry & Behavioral Medicine, Wake Forest University School of Medicine, Winston-Salem, NC, United States; ^7^Menninger Department of Psychiatry and Behavioral Sciences, Baylor College of Medicine, Houston, TX, United States; ^8^Department of Neurology, University of Rochester, Rochester, NY, United States

**Keywords:** Tourette, TIC, stigma, discrimination, scale, qualitative, quantitative

## Abstract

**Introduction:**

Persistent Tic Disorders such as Tourette Syndrome are common neurodevelopmental disorders that are highly stigmatized. Many individuals with Persistent Tic Disorders experience peer rejection, loneliness, and self-stigma. Experiencing stigmatization during childhood can influence the persistence of moderate-to-severe tics later in life. Additionally, these factors have been associated with increased suicidal ideation, suicide attempts, and psychiatric symptom severity. There is a need for interventions to reduce stigma and stigmatization in Persistent Tic Disorders. Before developing cost-effective interventions to mitigate stigma’s profound downstream health impacts, a reliable measure of stigmatization must be created. The overarching goal of this research is to develop and validate the Tourette Discrimination-Stigmatization (TD-STIGMA) Scale.

**Methods:**

This paper presents the study protocol for developing and validating the TD-STIGMA Scale. The study is designed as a mixed methods study to develop the TD-STIGMA scale and evaluate its psychometric properties. The study uses a phased approach: (1) collection of narrative and thematic content data through in-depth qualitative interviews of stakeholders, (2) development of a novel TD-STIGMA self-report scale using the Delphi Method based on these results, and (3) completion of analyses to determine the scale’s psychometric properties (confirmatory factor analysis, convergent, known-group, criterion validity, and test–retest reliability).

**Discussion:**

This project will result in a personalized approach to stigma measurement about youth and young adults with Persistent Tic Disorders, which to date does not exist. There are several limitations. Comorbidities or spiritual or cultural beliefs may affect perceptions of stigma and are not directly assessed in this study. We will utilize institutional resources for community outreach to purposefully sample underrepresented minorities who may be at disproportionate risk of adverse outcomes. However, this may not be fully representative of the generalized tic population. The study team will be purposeful in maintaining participant engagement for study retention. Lastly, participants from a tertiary referral center may not fully represent the generalized tic community. However, we hope our broad recruitment strategy and virtual study visits will facilitate a diverse and inclusive sampling of the patient population.

## 1 Introduction

Persistent Tic Disorders (PTD), including Tourette Syndrome (TS), are developmental neurobiological disorders characterized by multiple motor and vocal tics present for at least 1 year. PTD affects 1.4 million US children, adolescents, and adults (Tinker et al., 2022). Neuropsychiatric conditions co-occur in 90% of individuals with PTD/TS. PTD/TS and its co-morbidities, including attention-deficit hyperactivity disorder (ADHD) (Comings and Comings, 1987), obsessive-compulsive disorder (OCD) (Hirschtritt et al., 2015), and anxiety (Martino et al., 2017), impact how individuals interact with others and their environment at home, school, work, and in social activities.

Individuals with PTD/TS (ITS) experience many stressors related to their diagnosis, which can contribute to poor mental, physical, and functional outcomes. The lack of general knowledge and inaccurate beliefs of TS promotes an environment where ITS are often misunderstood (Lee et al., 2016, 2019; Ludlow et al., 2016, 2022; Ben-Ezra et al., 2017; Edwards et al., 2017; Lemelson and Tucker, 2017; Malli and Forrester-Jones, 2017, 2022; O'Hare et al., 2017; Schneider et al., 2018; Lee and Park, 2019; Fernández de la Cruz et al., 2021; Rodin et al., 2021; Stofleth and Parks, 2022; Stacy et al., 2023). Some ITS resort to suppressing their tics, concealing their diagnosis, or avoiding situations to prevent unwanted attention. Motivating factors for these behaviors include maintaining social normalcy and peer acceptance (O'Hare et al., 2015; Lee et al., 2016, 2019; Malli et al., 2019; Soós et al., 2022). Unfortunately, many ITS experience peer rejection (Lee et al., 2016, 2019, 2020; Lemelson and Tucker, 2017; O'Hare et al., 2017; Malli et al., 2019; Rodin et al., 2021; Stiede et al., 2021; Soós et al., 2022; Stofleth and Parks, 2022), the act of being labeled by others or *stigmatization* (O'Hare et al., 2015, 2016; Lee et al., 2016, 2019, 2020; Wadman et al., 2016; Edwards et al., 2017; Lemelson and Tucker, 2017; Jackson et al., 2019; Malli et al., 2019; Charania et al., 2022; Iyanda, 2022; Malli and Forrester-Jones, 2022; Mataix-Cols et al., 2022; Soós et al., 2022; Stofleth and Parks, 2022; Vermilion et al., 2022), loneliness (Lee et al., 2016, 2019; O'Hare et al., 2017; Malli et al., 2019; Rodin et al., 2021), and internalizing the negative stereotypes or *self-stigma* (Eaton et al., 2016; Hanks et al., 2016; Liu et al., 2017; Silvestri et al., 2017; Lee et al., 2019, 2022; Malli et al., 2019; Malli and Forrester-Jones, 2022). These experiences can influence the persistence of moderate-to-severe tics (Groth, 2018) and tic severity (Shiu et al., 2023). Additionally, while already higher risk than the general population (Comings and Comings, 1987; Cheung et al., 2007; Hirschtritt et al., 2015; Storch et al., 2015; Johnco et al., 2016; Martino et al., 2017; America TAO, 2022), studies show distress in ITS increases the risk of suicidal ideation (Van Orden et al., 2010; Yanos et al., 2010), suicide attempts (Fernández de la Cruz et al., 2017), psychiatric symptom severity (Johnco et al., 2016), and leads to poorer overall health (Fernández de la Cruz and Mataix-Cols, 2020).

Although research in this critical area is increasing, little is understood about why PTD/TS stigmatization persists despite decades of patient advocacy (Andersen, 1986; America TAO, 2022). Additionally, evidence-based interventions to reduce stigmatization and promote well-being in PTD/TS are lacking. However, a reliable measure of stigmatization must be created before developing cost-effective interventions to mitigate stigma’s profound downstream health impacts.

### 1.1 The social-ecological model of stigma and stigmatization

Stigmatization refers to labeling or linking to undesirable features, which creates beliefs that a person is fundamentally different (Andersen, 1986; Link and Phelan, 2001). Justifications are constructed for these stigmatizing actions toward others (Link and Phelan, 2001). Stigmatization can range from microaggressions, biases, and purposeful exclusion, while discrimination (also called enacted or experienced stigmatization) (Fernandes et al., 2011; Brohan et al., 2013) can be overt. The National Alliance on Mental Illness argues that the scope of stigmatization needs to encompass discrimination to adequately represent the negative impact of stereotypes these individuals experience (Sue Abderholden, 2019). Otherwise, it is easy to overlook the more distal behavioral effects when assessing the impact of stigma (Link and Phelan, 2001).

**Figure 1:**
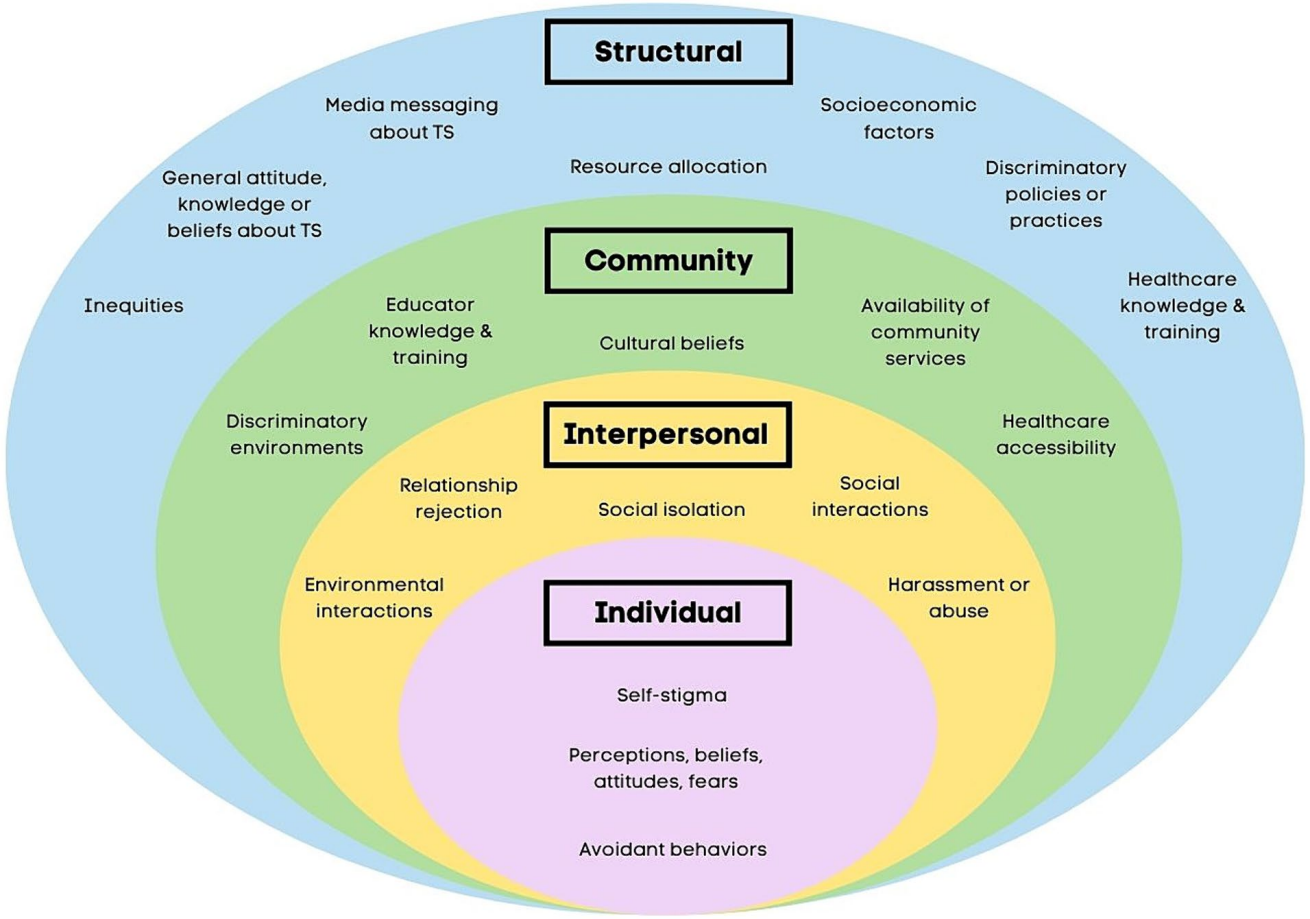
Social-ecological model of stigmatization in Tourette Syndrome.

However, stigmatization depends on social-ecological factors that create and perpetuate the labeling of stigma in the first place (Link and Phelan, 2001). The social-ecological model (SEM) has been used to model stigmatization in several other healthcare populations (Parker and Aggleton, 2003; Baral et al., 2013; White Hughto et al., 2015; Stangl et al., 2019). A SEM-based approach (Figure 1) derived from prior medical models of stigma (Parker and Aggleton, 2003; Baral et al., 2013; White Hughto et al., 2015; Stangl et al., 2019; Michaels, 2022) can help delineate factors contributing to stigmatization in PTD/TS. According to Link and Phelan’s theory, interventions that only target one mechanism leave the broader context untouched and are likely to fail (Link and Phelan, 2001). This is especially important in the context of interventions geared to prevent stigmatization. The SEM-based approach guides the development of a disorder-specific, comprehensive, relevant framework (Link and Phelan, 2001; White Hughto et al., 2015; Stangl et al., 2019). Globally, programs have been developed to diminish mental health-related stigmatization. However, stigma-related research remains limited by a lack of direct stigma measurements, limited validated youth measures (McKeague et al., 2015; Kaushik et al., 2017), proxy reports (Conelea et al., 2011), and non-validated questionnaires (Debes et al., 2010).

### 1.2 The necessity of a TS-specific measure

To face hardships, ITS requires *resilience* by harnessing resources to sustain well-being. The National Institute of Health (NIH) announced well-being as a 2023 funding priority (Mroczek et al., 2015), recognizing the potential impact of interventions focusing on well-being to improve health outcomes (Mroczek et al., 2015; Pressman et al., 2019; Villanueva et al., 2021). The NIH established a cohesive framework for well-being, defining *emotional well-being* as “*how positive an individual feels generally and about life overall. It includes experiential features (emotional quality of experiences) and reflective features (life satisfaction, meaning, and self-efficacy) within the context of culture, life circumstances, resources, and life course*” (Park et al., 2023). Existing interventions promoting well-being have shown sustained improvements in well-being and quality of life in cancer, type 1 diabetes, depression, and HIV/AIDS (Storch et al., 2012; McGuire et al., 2015; Cheung et al., 2018; Addington et al., 2019; Salsman et al., 2020; Leuchter et al., 2023; Moskowitz et al., 2023; Salsman et al., 2023). ITS need the opportunity to benefit from targeted, supportive interventions to enhance their well-being and resilience.

While evidence-based treatments exist, these focus on tic reduction and management. Barriers, including geography, availability, and the financial burden, make these treatments impractical or inaccessible for many (Pring et al., 2023). Stigma, motivation, and fear of making tics worse impact participation and success of treatment. Two pilot interventions incorporated positive coping strategies into their programs with preliminary reductions in tic-related impairment and improved quality of life (Storch et al., 2012; McGuire et al., 2015; Heijerman-Holtgrefe et al., 2021, 2022; BT-Coach, 2017). However, coping skills were not the primary intervention focus. Although these interventions reduced symptoms and impairment, the absence of impairment does not equate to well-being (Anderson, 1995; Constitution of the World Health Organization, 2005). It is critical to develop interventions that allow ITS to thrive despite their symptoms and foster their well-being.

When considering the theoretical framework for a PTD/TS-specific intervention that helps reduce psychological distress and promote well-being, stigmatization plays an integral role. However, there is no standard way to measure stigmatization in ITS. We postulate when ITS are stigmatized, they come to see their identity as threatened and unmanageable, which in turn adversely affects their subsequent mental, physical, and functioning. Malli reported when PTD/TS is perceived as self-defining, individuals express more disadvantages, anger, self-stigma, grieving the loss of their old self, and a deprived sense of normalcy (Malli et al., 2019; Malli and Forrester-Jones, 2022). Conversely, if they see their identity as protected and manageable in this framework, their outcomes will be better. As such, some adults believed PTD/TS provided the opportunity for self-development and search for greater meaning, allowing emotional strength and personal growth (Lee et al., 2016, 2019; Malli et al., 2019).

This conceptual framework is informed by the Transactional Model of Stress and Coping (Folkman, 1984) and Identity Threat Models of Stigma (Crocker et al., 1998; Berjot and Gillet, 2011), where stigmatized disorders, such as PTD/TS, include a primary appraisal of social or personal identity as individuals react distinctly different to identity-appraising and non-identity appraising situations. The secondary appraisal occurs when the ITS assess if they have the resources to cope with the situation.

If ITS have the resources to sustain well-being (resilience) in the face of adversity, does that change mental, physical, and functional outcomes? Antecedents such as social determinants of health, cultural and societal stereotypes, social support, and social resources are not directly modifiable without broad-scale interventions (Folkman, 1984; Berjot and Gillet, 2011). However, modifiable factors include how (digital, individual) and what (coping, stigma sensitivity) resources are developed and delivered. Additionally, intervening at either at the identity-appraisal or resource-appraisal stage can both be effective (Liu et al., 2021), but these are age- and context-sensitive (Panter-Brick and Leckman, 2013). The early challenges ITS experience can alter brain structure and function (McEwen, 2007; Karatsoreos and McEwen, 2011; Karatsoreos and McEwen, 2013) through epigenetic changes resulting in long-term consequences, including impacting their mental health outcomes (Green et al., 2010; Kessler et al., 2010). This overall process, *biological embedding* (Hanson and Gluckman, 2008; Shonkoff et al., 2009; Hertzman and Boyce, 2010), is most influential during periods of brain development such as early childhood and adolescence (Karatsoreos and McEwen, 2013) when tics are also beginning and peaking (Leckman et al., 1998; Freeman et al., 2000; Hirschtritt et al., 2015). Targeting interventions during these periods will have the greatest impact (Yousafzai et al., 2013).

Intervention development relies on the ability to test the conceptual framework and reliably measure stigma. Therefore, having a PTD/TS-specific measure is necessary. Existing measures have not been widely used nor studied in the youth population (Stuart, 2005; Kanter et al., 2008) nor address the full spectrum of PTD/TS-relevant stigmatization (Cella et al., 2012; Lai et al., 2012; McKeague et al., 2015; Kaushik et al., 2017). The overarching goal of this research is to develop and validate a PTD/TS-specific stigma measure, the Tourette Discrimination-Stigmatization Scale (TD-STIGMA), so we can test our conceptual framework for a wellbeing intervention in PTD/TS. This paper presents the study protocol for developing and validating the TD-STIGMA. The aims of this study are: (1) engaging with PTD/TS stakeholders to identify perceived stigmatization, experienced discrimination, and the need for targeted interventions in youth and young adults with PTD/TS, (2) to develop a clinical measure of stigmatization and discrimination using stakeholder input, and (3) to evaluate psychometric properties of the TD-STIGMA scale. The proposed research aims will inform the future development of a working clinical model and behavioral intervention that improves downstream mental, physical, and functional outcomes in ITS.

## 2 Methods

### 2.1 Setting

This study occurs at Wake Forest University School of Medicine (WFUSM), a tertiary facility in Winston-Salem, North Carolina. WFUSM serves as the academic core of Advocate Health, the nation’s fifth-largest nonprofit integrated health system in the United States. The southeast region has over 2,500 eligible English-speaking patients between 8 and 30 years old with diagnoses of PTD/TS. The southeast regional catchment area includes North Carolina, South Carolina, Virginia, Tennessee, and West Virginia. This study is registered under Clinicaltrials.gov NCT05696769 and the Open Science Framework https://doi.org/10.17605/OSF.IO/EX52G.

### 2.2 Participants

This study focuses on three specific cohorts: (1) youth and young adults with PTD/TS, (2) supporters, which include parents, caregivers, partners, or significant others, and (3) medical providers and advocates. Inclusion criteria for the ITS include a physician or neuropsychologist-confirmed diagnosis of PTD/TS based on the Diagnostic and Statistical Manual for Mental Disorders – Fifth Edition (DSM-V), English speaking, and between the ages of 8–30 years old. Exclusion criteria include a diagnosis of intellectual disability, active psychosis, or any other condition that, in the Principal Investigator’s opinion, would limit the participant’s (or parent’s) ability to understand study measures. Inclusion criteria for the supporter cohort include caring or having a relationship for/with a youth and young adults with PTD/TS for at least 1 year and being English-speaking. Inclusion criteria for the medical providers and advocates cohort include caring for a youth and young adults with PTD/TS and speaking English.

### 2.3 Overall study design

The study is designed as an exploratory sequential mixed methods study to develop the TD-STIGMA scale and evaluate its psychometric properties. The study uses a phased approach: (1) collection of narrative and thematic content data through in-depth qualitative interviews of PTD/TS stakeholders, (2) development of a novel TD-STIGMA self-report scale using the Delphi Method based on these results, and (3) completion of analyses to determine the scale’s psychometric properties (confirmatory factor analysis, convergent, known-group, criterion validity, and test–retest reliability). We report how we determined our sample size, all data exclusions, all manipulations, and all measures in the study.

#### 2.3.1 Recruitment

Eligible patients in the southeast region of our healthcare enterprise will be identified through the Translational Data Warehouse (TDW). The TDW aggregates data from the electronic health record and other clinical systems for research purposes. The TDW includes information on more than 6.6 million unique patients and over 270 million encounters. The southeast region encompasses five states: North Carolina, South Carolina, Virginia, Tennessee, and West Virginia. The southeast region has over 2,500 eligible English-speaking patients between 8 and 30 years old with diagnoses of PTD/TS. Demographic data shows 73% are male, 27% are female, 83% are White, 9% are Black, and 8% are other URM (American Indian, Alaskan Native, Asian, Other Pacific Islander, and Latin American or Hispanic). Black and Latino have half the rate of TS compared to White individuals (Blumberg et al., 2007) yet increased risk for marginalization and poorer outcomes (Luthar, 1991; Brooks, 2006; Masten, 2011).

Participants will be recruited through normal patient flow at Atrium Health Wake Forest Baptist specialty clinics, referral networks, direct mailers, and regional TS chapters. Additional participant identification and recruitment will occur through the *Be Involved* network, a community-facing website that helps patients, community members, and healthy volunteers to find study opportunities in the region. We will partner with Maya Angelou Center for Health Equity (MACHE) Integrating Special Populations Program of the Wake Forest Clinical and Translational Science Institute (CTSI) to facilitate purposeful enrollment and retention of those are at disproportionate risk of adverse outcomes.

#### 2.3.2 Sample size

Sample sizes in qualitative studies are guided by whether there will be sufficient information power based on five study dimensions including specific aims, sample specificity, use of established theory, quality of dialogue, and analysis strategy. While the scope of our study is broad, enrollment of the target population, strong communication, theoretical framework, and analytical strategy guides our sample size enrollment projections for the qualitative interviews. Based on this information power model (Malterud et al., 2016) the goal for semi-structured interviews is 20 individuals with PTD/TS (ages 8–17, *n* = 10; ages 18–30, *n* = 10), five supporters, and ten providers/advocates. We will attempt to reach data saturation in the PTD/TS interview groups based on the expected sex distribution of PTD, 4:1 M:F (Leckman et al., 1998; Freeman et al., 2000; Garris and Quigg, 2021). These estimates allow a large enough sample to gather new and rich information but still small enough to gather detailed data (Sandelowski, 1995). If valuable information is still being identified after 80% of interviews, we will consider adding participants (Lincoln and Guba, 1985; Malterud et al., 2016) until nothing new is learned (Saunders et al., 2018). Given the relative rarity of PTD/TS and that the domains in confirmatory factor analysis have been identified in prior literature (Ritsher et al., 2003; Cella et al., 2012; Lai et al., 2012), for determining the psychometric properties of the TD-STIGMA, 100 individuals with PTD/TS (ages 8–17, *n* = 50; ages 18–30, *n* = 50) will be enrolled.

#### 2.3.3 Development of the demographic domains

Demographic questions have been compiled from several validated scales relevant to this study, including the National Survey of Children’s Health (NSCH) (National Survey of Children’s Health, 2021), the Center for Disease Control (CDC) Youth Risk Behavior Surveillance System (YRBSS) (Youth Risk Behavior Surveillance System, 2019), and the CDC National Health Interview Survey (NHIS) (Control CfD, 2022). Survey questions were not modified to be able to compare our sample to the national data sets. Demographic questions were chosen to align with the domains in the interview guides. These will be completed in REDCap (Research Electronic Data Capture) before the qualitative interview. All project materials can be accessed at https://doi.org/10.17605/OSF.IO/AN5T3.

#### 2.3.4 Development of exploratory questionnaire

An initial set of 44–47 stigma questionnaire items was developed using domains of interest, clinical expertise, and gaps in the literature and modeled from validated stigma scales. The Questionnaire on Anticipated Discrimination (QUAD) (Gabbidon et al., 2013), Discrimination and Stigma Scale (DISC) (Bakolis et al., 2019), PaedS (Kaushik et al., 2017), PMHSS (McKeague et al., 2015), and specific questions of the Internalized Stigma of Mental Illness (ISMI) (Ritsher et al., 2003) were used as models for stigma questions. Items were created to include perceived and experienced stigmatization of the individual with PTD/TS at each level of the SEM: individual, interpersonal, community, and structural. Examples of selected questions are provided in Table 1.

**Table 1:** Selected examples from exploratory questionnaire TD-STIGMA questions – youth and young adults.

**SEM level**	**Question**	**Perceived stigmatization?**	**Experienced stigmatization?**
Structural	How are you treated at a doctor’s office, hospital, or medical facility because of your tics?	X	X
Structural	What are the biggest misunderstandings people have about your tics?	X	
Structural	Do you feel your doctors understand your tics?	X	
Community	How does having tics impact your experiences in public?	X	X
Community	What barriers have you encountered to getting the treatments recommended by your doctor for your tic disorder?	X	X
Community	Have you been treated differently by your school because of your tics?	X	X
Community	What impact does your tic disorder have on your ability to work?	X	X
Interpersonal	How does having tics impact relationships?	X	X
Interpersonal	What does having Tourette Syndrome or tics mean to your family?	X	
Individual	What does having a tic disorder mean to you?	X	
Individual	How does having tic disorder make you feel as a person?	X	

The exploratory questionnaire covers several domains, including experienced and perceived stigmatization at each SEM level, positive psychosocial factors (*In what ways do having tics make you stronger? What has been most helpful for you in coping with your tics?*), contributing or confounding stigmatization risk factors (*gender identity, sexual identity for adults, sociodemographic* var*iables*), and intervention priorities (*What do you feel would be most helpful in addressing some of the challenges you have mentioned?*)

There are eight sections on the exploratory questionnaire including how tics make people feel, experiences at school, relationships with others, experiences at jobs, experiences with medical care, in public or at home, and priorities for interventions. The questions are adapted to the age appropriateness and type of the participant. This resulted in four versions of the exploratory questionnaire: (1) youth (8–17 years), (2) young adult version (18–30 years), (3) supporters (parents, caregivers, partners), and (4) medical providers or advocates. Several study members reviewed and revised the questionnaires for flow, content, and length. There are 24–30 items developed referencing PTD/TS, depending on the version, and four items about the survey experience.

#### 2.3.5 Eligibility visit

For individuals with PTD/TS who are interested in the study, eligibility visits will be scheduled in person or via secure video with the study physician or neuropsychologist to confirm eligibility. If eligible, the Yale Global Tic Severity Scale (YGTSS) with tic inventory will also be completed (Storch et al., 2005).

#### 2.3.6 Qualitative interviews

The qualitative interviews will be conducted individually with the study team and members of the Qualitative and Patient-Reported Outcomes (Q-PRO) Shared Resource. All interviews will be conducted via in-person or secure video. Interviews will be recorded to allow audio transcription for qualitative analysis. As mentioned previously, the exploratory questionnaire serves as the interview guide with questions adapted for age appropriateness and participant type. Topics covered include how tics make people feel, experiences at school, relationships with others, experiences at jobs, experiences with medical care, in public, or at home, and priorities for interventions.

Youth will be given time to answer sensitive questions confidentially. Youth will be asked about gender identity (for 12 and up), home environment, bullying, self-harm, risky behaviors, and their personal experiences. These answers will be confidential unless there is a concern that the there is a safety risk to themselves or others. At the end of the interview, participants will provide feedback regarding ease of completion, content, and completion time. Interviews are estimated to take 60–90 min to complete. Breaks will be provided as needed, and participants will be compensated for their time.

#### 2.3.7 Qualitative analysis

Qualitative analysis will include transcribing verbatim and de-identifying all audio-recorded interviews in ATLAS.ti software. The study team will analyze the interviews using thematic content analysis practices (Green and Thorogood, 2018) to identify emerging themes and subthemes. Two study team members will develop a draft codebook including inductive and deductive codes, which will be reviewed and edited by the study team accordingly. Two study team members will then independently code the transcripts, meet regularly to discuss, and resolve discrepancies and reach a consensus. As needed, the PI (Principal Investigator) will serve as a third coder for unresolved discrepancies. Reports will be run for each code, and data will be reviewed within single codes or combination of codes to identify patterns and themes with the assistance of Q-PRO.

#### 2.3.8 Feedback process

**Figure 2:**
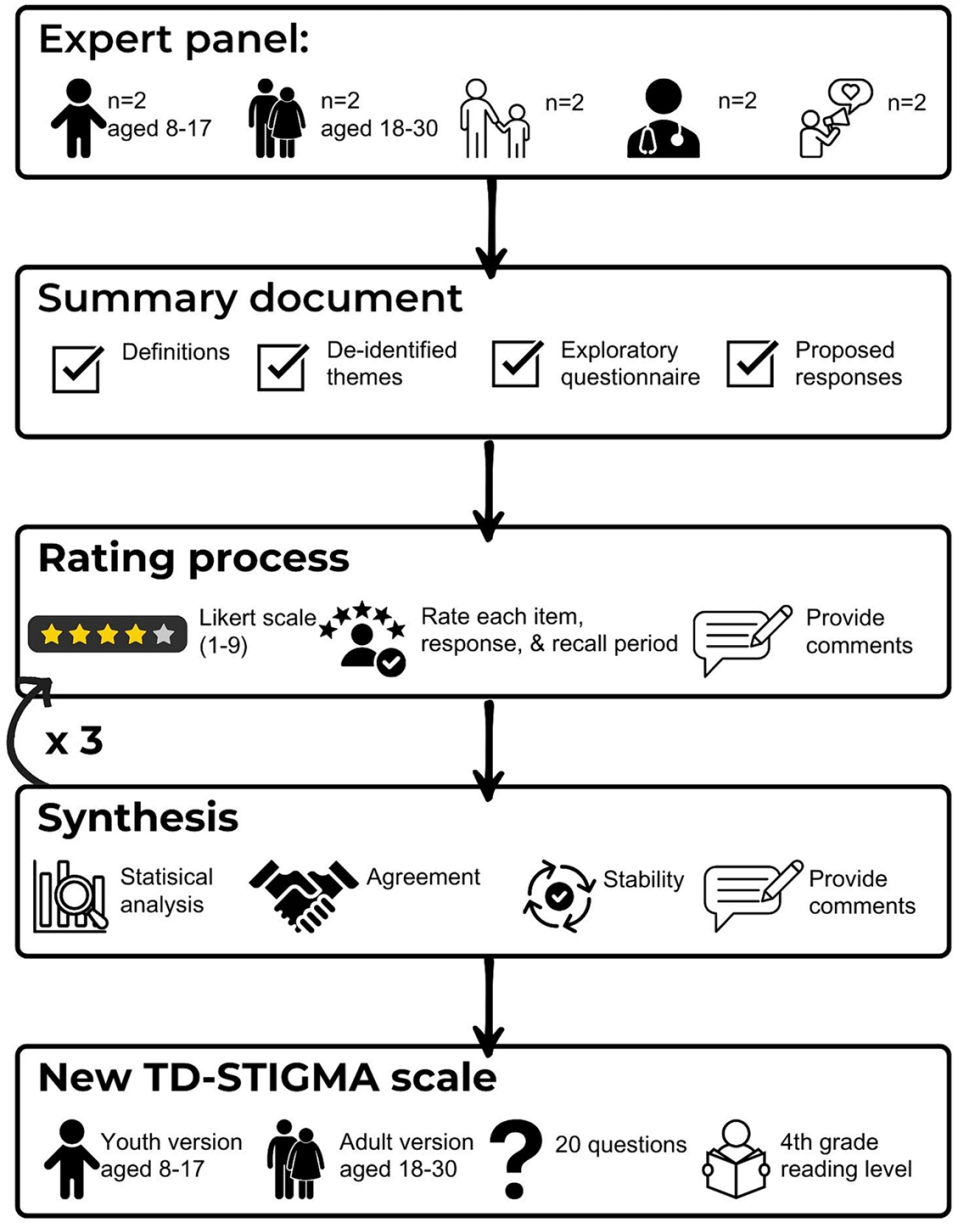
TD-STIGMA feedback process.

A total of 10 participants from Aim 1 will be invited to be the expert panel (two individuals with PTD/TS ages 8–17 years, two individuals with PTD/TS ages 18–30 years, two caregivers, two medical providers, and two advocates). A summary of the feedback process can be seen in Figure 2. We will use a holistic review to ensure diverse representation among the panelists. The main selection criteria will be the diversity of the panel member’s background or specialty, practice setting if applicable, geographic representation, minority representation, and absence of conflicts of interest. Panelists will complete the Delphi method through REDCap to maintain anonymity, minimize group conformity, and increase panelist comfort in providing their opinions.

A concise list of indications and definitions will be provided to begin the Delphi method. A de-identified, alphabetized summary document of thematic content and narrative content analysis from Aim 1 will be securely emailed by blind-copying the panel to maintain anonymity and minimize bias. An initial indication matrix will be created from the exploratory questionnaire and proposed response options based on the Rand UCLA Appropriateness Method (Fitch, 2001). The panel will rate items on a Likert scale of 1–9 (1 = extremely inappropriate, 5 = uncertain, 9 = extremely appropriate). Each item will be rated separately for ages 8–17 and 18–30. The panel will also be asked to rate the proposed recall period and instructions.

During the first round of ratings, panelists will rate each item as described above and can provide open-ended survey responses on each question. Statistical analysis will synthesize the responses and content analysis of the open-ended responses will be conducted to identify any additional relevant information. The Rand UCLA criteria for agreement will be used. Disagreement is indicated by at least three panelists rating the indication in each extreme of the 9-point Likert scale.

During additional rounds, the panel will be provided with their responses, the frequency of the panel responses, and the panels’ anonymous comments. The individual rater will re-rate each item. We anticipate three rounds; however, additional closing criteria include achieving agreement and stability of responses for two rounds (Sekayi and Kennedy, 2017). The outcome of this process will be a close-ended TD-STIGMA scale. The Flesch Reading Ease (Flesch, 1948) score and Flesch–Kincaid Grade (Thomas et al., 1975) level will be used to assess questionnaire readability. We will aim for a fourth-grade reading level.

After the exploratory questionnaire item pool has been developed, the selection of items for quantitative analysis will follow an iterative process of expert and stakeholder reviews based on the Delphi Method. The comprehensive review of the items aims to establish face and content validities of the scale. We expect to eventually include 20 items for each version (young adult and youth) in the finalized questionnaire on *N* = 100 participants (*N* = 50 young adult and *N* = 50 youth).

#### 2.3.9 Second interviews

The same recruitment methods will be used for the second interview. Participants with PTD/TS who participated in Interview 1 will be invited to participate again. Interviews will be scheduled in person or by secure video to complete the clinician administered YGTSS and inventory. The remainder of the study visit includes self-report and parent-proxy companion measures entered REDCap. The companion measures will be included to establish the psychometric properties of the TD-STIGMA scale. Participants will be compensated for their time.

##### 2.3.9.1 Companion measures

The clinician-rated (YGTSS) is the gold-standard measure of tic severity. It is a clinician-administered, semi-structured inventory that measures tic severity and impairment over the past week. Motor and vocal tics are rated separately for their number, frequency, intensity, complexity, and interference on a 0–5 Likert scale. The total is summed to reflect motor tic severity (0–25), phonic tic severity (0–25), or combined tic severity (0–50). A single question rates tic-related impairment (0–50). The YGTSS displays excellent internal consistency, reliability, and convergent and divergent validity (Storch et al., 2005).

Quality of Life in Neurological Disorders (Neuro-QoL™) (Perez et al., 2007; Cella et al., 2011) instruments were developed through a collaborative, multi-site research initiative sponsored by the National Institute for Neurological Disorders and Stroke to construct psychometrically sound and clinically relevant health-related QoL measurement tools for individuals with neurological conditions. Through Neuro-QoL, the pediatric (Lai et al., 2012) and adult (Cella et al., 2012) stigma question bank is utilized. These are brief, reliable, valid, and standardized QOL assessments applicable across neurologic conditions. The adult bank has 24 questions for self-report, and pediatric (Lee et al., 2016, 2019; Ludlow et al., 2016, 2022; Lemelson and Tucker, 2017; Malli and Forrester-Jones, 2017, 2022; O'Hare et al., 2017; Lee and Park, 2019; Rodin et al., 2021) has 18 questions for self or parent report.

The Internalized Stigma of Mental Illness Inventory (ISMI) includes 29 items with five subscales assessing alienation, stereotype endorsement, discrimination experience, social withdrawal, and stigma resistance. Data analysis showed high internal consistency, test–retest reliability, and construct validity (Ritsher et al., 2003) as there are no validated discrimination measures in youth; adapted versions interchanging the word “mental illness” with persistent tic disorders are utilized for adult self-report and parent proxy reports. While changing the terminology and the age of use poses concerns for validity, in this study, we will gather proof of principle and preliminary data in preparation for future larger projects.

### 2.4 Scale development method

Descriptive statistics will be used to examine TD-STIGMA item-level scores for detecting ceiling/floor effects, outliers, normality, and other problematic distributional characteristics. Such initial screening of the items may result in removing items that do not perform well (e.g., strong ceiling effect).

Following guidelines from the modified Consensus-based Standards for selecting health Measurement Instruments (Prinsen et al., 2018), we plan to evaluate the scale’s psychometric properties along several aspects of validity. The assessment of face and content validities is described in the section Feedback Process. Confirmatory factor analysis (CFA) will also assess the structural validity for the hypothesized domains-- structural, community, interpersonal, and individual levels of stigma and discrimination (Figure 1). We do acknowledge the smaller sample size. However, given the relatively rare disease and that the domains in CFA have been identified in prior literature we felt it was appropriate. A fifth protective domain will include protective measures against stigma and discrimination. Missing values for the CFA will be treated as missing-at-random, and the full information maximum likelihood method, in which only those variables observed for a case would enter the estimation procedure, will be used to maximize efficiency. We will use multiple commonly accepted goodness-of-fit indexes to evaluate if the *a priori*-determined factor structure is valid (Hu and Bentler, 1999). We also plan to conduct a bifactor analysis (Gibbons and Hedeker, 1992). Because most of the items in TD-STIGMA will be adapted from validated instruments, we expect only minor adjustments (e.g., item removal) will be needed to establish the structural validity for the domains in TD-STIGMA. The subscale score will be formed from the sum score of the items under the subscale, and the unweighted sum of subscales will form the overall score.

We expect missing values to be minimal. If the proportion of missing items exceeds 50%, the participant will not receive a score and will be excluded from all analyses. Internal consistency will be evaluated using Cronbach’s alpha at the item and the subscale/scale level. The intraclass correlation will assess test–retest reliability by retesting a subset of clinically stable participants (*n* = 20) 2 weeks after test administration. We expect the test–retest intraclass correlation coefficient to be above 0.70 (Paiva et al., 2014). Items may be removed if that criterion is not achieved. Both convergent validity and known-group validity will be evaluated. Convergent validity will be evaluated by assessing the correlation coefficients between the subscale/scale of TD-STIGMA and the companion measures. We plan to use the criterion based on the ISMI for assessing criterion validity. We will use a cutoff of 2.5 on the ISMI scale (Ritsher et al., 2003; Boyd et al., 2014; Bengochea-Seco et al., 2018), of which the total score range is 0 to 4, 4 being the worst, to indicate the presence of stigma. We will compare the means of TD-STIGMA of the stigma and the non-stigma groups using a t-test. We hypothesize that TD-STIGMA is higher in the stigma group identified by ISMI. The analyses will use the SAS 9.3 statistical software and Mplus v8.

### 2.5 Safety protocol

There may be discomfort in discussing some of the information, or it may be difficult for caregivers to hear. If a participant becomes distressed or triggers a reportable event, the study team will follow the outlined procedures as appropriate. Reportable events include positive suicidal ideation, suicidal attempt, self-harming behaviors, or another mandated event.

### 2.6 Confidentiality, privacy, and data management

All appropriate measures will be taken to ensure confidentiality including restricted access to secure study files, de-identified transcripts, and using unique study identifiers. We will not reuse the recordings. The audio or video will be uploaded into a secure study folder. Data for this initiative will be entered into a REDCap database. Following data collection, the subject identifying information is destroyed 6 years after the closure of the study, consistent with data validation and study design, producing an anonymous analytical data set. No reference to any individual participant will appear in reports, presentations, or publications that may arise from the study.

## 3 Discussion

There are several limitations. Perceptions of stigma may be influenced by comorbidities and spiritual or cultural beliefs. As part of a larger study, we will systematically assess participant’s anxiety, depression, and positive affect. To ensure a narrow scope, feasible study, and limited survey burden, self-reported diagnoses of all co-occurring conditions will be reported and will be more systematically explored in future studies. We will utilize MAHCE for community outreach to purposefully sample marginalized participants who may be at disproportioned risk of adverse outcomes. However, this may not be fully representative of the generalized population. These will be considered in future work with larger samples. Secondly, for most participants enrolled in qualitative interviews, there is a gap in study participation. The study team will work with MACHE to develop a strategy for maintaining participant engagement to maintain study retention. A study website and newsletter may be helpful in keeping participants up to date on the study’s progress. Lastly, participants from a tertiary referral center may not fully represent the generalized PTD. However, we hope our broad recruitment strategy and virtual study visits will facilitate a diverse and inclusive sampling of the patient population.

## 4 Conclusion

This project will result in a personalized approach to stigma measurement about youth and young adults with TS, which to date does not exist. We envision that such a measure will have implications for assessment and treatment. Quantifying stigma at the start of treatment will help understand potential clinical barriers in the environment, which may be targets of behavioral interventions. Particularly addressing the need to promote well-being in ITS and, in turn, improve downstream mental, physical, and functioning. Additionally, evaluating the broader impact of stigma may help advocate for increased allocation of funding for resources, research, and education to mitigate stigmatization.

## Ethics statement

The studies involving humans were approved by Wake Forest University Health Sciences Institutional Review Board. The studies were conducted in accordance with the local legislation and institutional requirements. Written informed consent for participation in this study was provided by the participants' legal guardians/next of kin.

## Author contributions

JMM: Conceptualization, Funding acquisition, Investigation, Methodology, Project administration, Resources, Writing – original draft, Writing – review & editing. VR: Investigation, Project administration, Writing – original draft, Writing – review & editing. KP: Investigation, Writing – original draft, Writing – review & editing. SN: Project administration, Resources, Supervision, Writing – review & editing. KW: Conceptualization, Investigation, Project administration, Supervision, Writing – original draft, Writing – review & editing. RS: Methodology, Resources, Supervision, Writing – review & editing. LC: Investigation, Methodology, Resources, Writing – review & editing. JR: Conceptualization, Investigation, Methodology, Resources, Supervision, Writing – original draft, Writing – review & editing. SR: Conceptualization, Methodology, Resources, Supervision, Writing – review & editing. ES: Conceptualization, Resources, Supervision, Writing – review & editing. EI: Conceptualization, Formal analysis, Methodology, Resources, Supervision, Writing – original draft, Writing – review & editing. JWM: Conceptualization, Methodology, Resources, Supervision, Writing – review & editing.

## Glossary

PTD: Persistent tic disorders
TS: Tourette syndrome
ADHD: Attention-deficit hyperactivity disorder
OCD: Obsessive-compulsive disorder
DSSS: Depression self-stigma scale
ISE: The inventory of stigmatizing experiences
PaedS: The pediatric self-stigmatization scale
PMHSS: Peer mental health stigmatization scale
Neuro-QoL™: Quality of life in neurological disorders
TD-STIGMA: Tourette discrimination-stigmatization scale
SEM: Social-ecological model
WFUSM: Wake Forest University School of Medicine
DSM-V: Diagnostic and Statistical Manual for Mental Disorders – Fifth Edition
TDW: Translational data warehouse
MACHE: Maya Angelou Center for Health Equity
ISP: Integrating special populations
CTSI: Clinical and translational science institute
URM: Underrepresented minorities
REDCap: Research electronic data capture
NSCH: National Survey of Children’s Health
CDC: Center for Disease Control
YRBSS: Youth risk behavior surveillance system
NHIS: National Health Interview Survey
QUAD: Questionnaire on anticipated discrimination
DISC: Discrimination and stigma scale
ISMI: Internalized stigma of mental illness
YGTSS: Yale global tic severity scale
Q-PRO: Qualitative and patient-reported outcomes
CFA: Confirmatory factor analysis
